# Personalized Human Papillomavirus Vaccination for Persistence of Immunity for Cervical Cancer Prevention: A Critical Review With Experts' Opinions

**DOI:** 10.3389/fonc.2020.00548

**Published:** 2020-04-23

**Authors:** Ramadhani Salum Chambuso, George Rebello, Evelyn Kaambo

**Affiliations:** ^1^Division of Human Genetics, Faculty of Health Sciences, University of Cape Town, Cape Town, South Africa; ^2^MRC Unit for Genomic and Precision Medicine, Division of Human Genetics, Department of Pathology, Faculty of Health Sciences, University of Cape Town, Cape Town, South Africa; ^3^Department of Biochemistry and Medical Microbiology, School of Medicine, University of Namibia, Windhoek, Namibia; ^4^Division of Medical Virology, Department of Pathology, Faculty of Health Sciences, University of Cape Town, Cape Town, South Africa

**Keywords:** HPV vaccine, cervical cancer, vaccine adjuvant, host genetics, immune response, personalized vaccination

## Abstract

The development of cervical cancer has been shown to involve both viral and host factors. The host factors are those that determine the specific response to human papillomavirus (HPV) infection by the patient's immune system. The immune responses to vaccines have been shown to be influenced by polymorphisms in genes involved in innate and adaptive immunity. The specific genetic variants that may influence the immune responses to HPV vaccine which may contribute to persistence of immunity (POI) have not been widely studied yet. In order to address the question as to “is it right to vaccinate all children, and all with equal dose?” we have critically examined the knowledge of common immunogenetic and immunogenomic variations that may influence the HPV vaccine POI across various populations. We have also identified a number of specific research questions that need to be addressed in future research into host molecular genetic variations and HPV vaccine POI in order to afford life-long protection against the development of cervical cancer. This work informs future insights for improved HPV vaccine designs based on common host molecular genetic variations.

## Introduction and Background

Vaccines have revolutionized public health by preventing mortality and reducing morbidity for millions of individuals (1). For example, infections like *Haemophilus influenzae* type b (Hib) and *Neisseria meningitidis* alone, are estimated to cause 340,000 episodes of severe Hib in the year 2015, and there are about 500,000–1,200,000 invasive meningococcal diseases occur each year worldwide (2, 3). Despite prior vaccination, over 150,000 Hib and 50,000 meningococcal cases resulted in deaths between 1990 and 2013 (4). Although there may be other reasons such as a possibility of non-vaccine serogroups, this “failure of vaccination” arises because the maintenance of specific antibodies is essential for life-time continuity of vaccine-induced immunological protection (5). Yet, there is a considerable variability in the magnitude and persistence of vaccine-induced immunity in different populations (5).

The population-level public health paradigm of “one size fits all” has been the norm in vaccine-preventable diseases (6). Introduction of childhood immunization with human papillomavirus (HPV) vaccine has resulted in apparent decrease of abnormal cervical lesions and HPV infection incidence in adulthood in some populations (7). While the HPV vaccine addresses the immune response to the virus, a focus on host molecular genetic variations in immune responses to the vaccine must also be considered. This is because long lasting serum antibodies to cervical HPV infection are essential for a life-long protection against the development of cervical lesions (8). Currently, there is very limited evidence on whether the development of abnormal cervical lesions will be permanently preventable in all women, despite demonstrable acute vaccine immunogenicity during HPV vaccination in different populations (9–11).

The goal of vaccination is to provide a life-time persistence of specific antibodies against HPV infection, primarily because the effectiveness and acceptability of the HPV vaccine would be greatly improved if the protection were sustained, without the need for repeated boosting throughout life. Currently, long-term follow-up studies evaluating increased effects of the booster doses of HPV vaccine are limited (12). There are a number of approaches to improve vaccine effectiveness: On the one hand, vaccine adjuvants, which facilitate increased and longer-lasting immunity in vaccines are crucial for the effectiveness of the HPV vaccine persistence of immunity (POI) (11, 13). On the other hand, immunogenetic and immunogenomic variations in host immune response genes are involved in directing CD4+T cell responses for a long-term HPV immunity. Variants encoding Toll-like receptors, human leukocyte antigen (*HLA*) molecules, cytokines, and cytokine receptors, have been associated with heterogeneity of immune responses to a wide range of vaccines (6, 14).

However, a number of factors have been implicated in determining host immune responses to vaccines, and the POI, these are: age, sex, ethnicity, microbiota, nutritional status, and infectious diseases (13, 15–19). Importantly, studies on vaccine immune responses in different ethnic groups have started to unpick the genetic components underlying persistence of vaccine-induced immunity (5, 20). Furthermore, the genome-wide association study (GWAS) approach has also provided considerable insights into the vaccines immune responses and host molecular genetic variations (5, 21, 22).

In order to assure individualized POI is provided by the HPV vaccine, host immunogenetic and immunogenomic profiling will be required in the future (23). This will advance the state of knowledge by revealing novel interactions between, and within, the immune system, individual genetics, and genomics in different population groups (23). In turn, this genetic knowledge will inform at both individual, and population levels in an “unblinded” genetic manner the following:

- The genetic identification of individuals, who are at increased risk for cervical HPV infection and cervical disease development (24).
- The identification of the genetic mechanisms behind the variations in HPV vaccine immunogenicity and effectiveness in different populations (25).
- The genetic prediction of potentially serious adverse events after HPV vaccination (23).
- Precision genetics methods to optimize the HPV vaccine dosing required for fully persistent protection in the practice of Vaccinology (26).

Herein we propose novel concepts in common host immunogenetic and immunogenomic variations in immune response genes that may impair HPV vaccine POI for cervical cancer prevention in only a small fraction of women in different populations. Moreover, we identify novel research gaps and research opportunities in host genetic variations, and the HPV vaccine POI for cervical cancer prevention. This work will inform future insights for improved personalized HPV vaccine designs based on common host molecular genetic variations.

## Common Host Molecular Genetic Variations and the Immune Responses in the regulation of HPV Vaccine Poi

In addition to the assessment of differences in the immune responses to HPV infection, polymorphisms in common host molecular genetic variations are of great interest. The investigation of host genetic susceptibility factors should also consider the following:

- Identifying susceptibility factors for HPV persistence (27).
- Identifying susceptibility factors for progression of cervical lesions to carcinoma (28).

**Figure 1:**
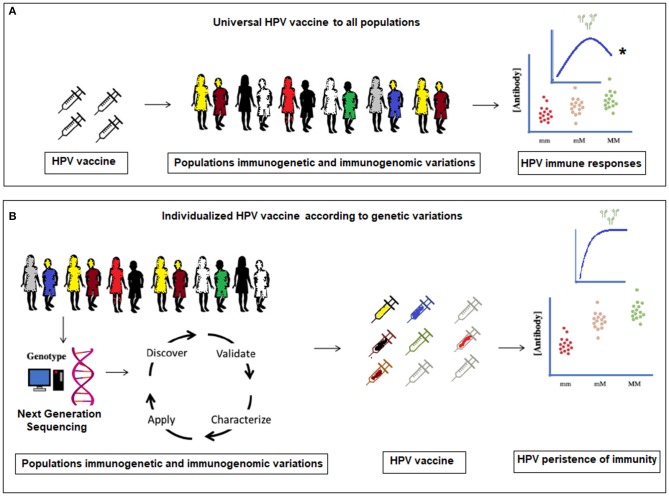
**(A)** Current universal HPV vaccination to all populations without considering molecular genetic variations. *The antibody levels plateau and a significant waning within a certain time-frame in **(A)**, is proposed to occur only in a small number of women in the vaccinated population. **(B)** Proposed individualized HPV vaccination according to specific population immunogenetic and immunogenomic profiling.

In this section, we assess the host immunogenetic and immunogenomic factors with generalizable effects on HPV vaccine immune responses (6) (Figure 1), while recognizing that there may also be other factors influencing the immune responses to the HPV vaccine, and the POI.

It is estimated that, “between 44 and 78% of the variations observed in antibody responses to vaccines are genetic in origin” (29, 30). Currently, we are not aware of any implemented healthcare approach that attempts to identify women whose HPV vaccine-induced immunity has waned and would benefit from additional immunizations or vaccine boosters. We propose to identify genetic markers for long-term HPV vaccine immune responses and to prove their clinical utility in the persistence of HPV immunity (31). Furthermore, Next-Generation Sequencing (NGS), either by whole genome sequencing (WGS) or whole exome sequencing (WES) can be used for host genetic profiling of a number of genetic risk factors, which may modify HPV vaccine POI, before the vaccination of a patient (29, 32). It is feasible that in the near future, the HPV vaccine POI for an individual patient could be predicted, and vaccine regimens be personalized, in order to maximize persistence of vaccine effectiveness throughout the life of the vaccinated individual.

Since cervical cancer is a complex disease, it is likely that genetic involvement consists of a large number of genes, each conferring different levels of risk (33, 34). Although it is difficult to study all these host immune response genes at the same time, distinct populations exist that may be informative in identifying such susceptibility genes, these are:

- Participants in HPV vaccine clinical trials can be examined in order to assess individual HPV vaccine heterogeneity of immune responses to the vaccine and its POI (35).
- Women with rapid-onset cervical disease can be examined to assess individual HPV exposures and the influence of molecular genetic factors (24, 36).
- Women with a high number of sexual partners (repeated exposure to oncogenic HPV infection), who do not develop cervical neoplasia can help to assess individual genetic factors for natural immunity to HPV persistent infection (37).
- Older women with a persistent oncogenic HPV infection who do not develop cervical neoplasia can be examined to assess individual genetic factors for natural immunity to HPV persistent infection (37).

Given the complexity in molecular genetic variations of the host and immune responses to HPV vaccine, future efforts will likely require the use of high-throughput genetic technologies, such as WES, to assess the polymorphisms of numerous immune response genes simultaneously, in a timely and accurate manner. This will enable comprehensive assessment of personalized host immune gene pathways rather than a limited number of candidate genes or markers.

## The Role of HLA Molecules as Immune Response Genes, and the Possible Effects of the HPV Vaccine Adjuvants on HPV Poi Attributable to Molecular Genetic Variations

Molecular genetic variations exert distinct influences on antigen-specific immune responses (29, 32). The role of HLA genes as regulators of immune responses may also depend on the nature of the immune responses induced by different antigens (38, 39).

However, the identification of causal variants within the HLA region is intrinsically difficult due to the complex polymorphisms found in this region. During HPV vaccination, the early immune response signals originate from differing abilities of classical HLA alleles to bind virus-like particles (VLPs). The HLA genes, through innate immunity, play an important role in the initiation process of producing antibodies against viral infection (29). HLA-associated vaccine failure has already been reported for several vaccines (14, 26, 40, 41) and non-HLA genes have also been shown to play a dominant role in antibody responses including total IgG production (5). Newport et al. (29), suggested that non-HLA genes exert a strong control on B-cell responses, whereas data from O'Connor et al. (5), in older population samples, suggest that HLA genes influence antibodies responses triggered in immunologically matured individuals. However, Fletcher et al. (42) showed that IFN-g responses to some vaccines (non-HPV vaccines), such as TB vaccine, are predominantly influenced by the HLA class II genes. A study by Leo et al. (43) had adequate power to dissect the HLA associations in a cervical cancer study. They found that “three HLA II haplotypes, HLA-DRB1^*^15/HLA-DQB1^*^0602/HLA-DQA1^*^0102,HLA-B^*^0702/HLA-C^*^0702, and HLA-DRB1^*^0401/HLA-DQA1^*^0301, were associated with increased risk for cervical neoplasia” after HPV infection.

**Figure 2:**
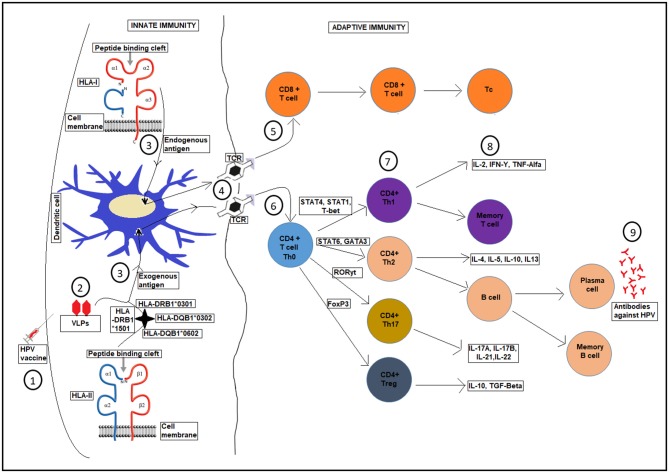
A schematic diagram showing *HLA* presentation of human papilloma-VLPs in the HPV vaccine, with respect to host *HLA* variations and the whole T-cell activation process for production of antibodies against HPV infection. Upon HPV vaccination (1), VLPs are introduced, (2). Different HLA genes present the HPV-VLPs to the dendritic cells as exogenous or endogenous antigens (3), which then go through a maturation process. The dendritic cells present the antigens on HLA class I or II molecules on the cell surface of adaptive immunity through T-cell receptor TCR (4), into CD8+ T-cells (5), and CD4+ T-cells (6) to become antigen specific effector T-cells for further processes (7 and 8) until the production of antibodies against HPV infection (9).

In innate immunity antigen-presenting cells (APCs), such as dendritic cells, internalize the HPV vaccine VLPs and display them on their surfaces with the help of major histocompatibility complex (MHC) genes (*HLA* class *I* and *HLA* class *II*) as endogenous or exogenous antigens, respectively (44). This triggers T cell immune responses in adaptive immunity via T cell receptors (TCR) that bind those antigens and differentiate into effector, or killer, T cells. These killer T cells mobilize the immune reaction against the VLPs peptides by the production of antibodies which act against HPV infection (44) (Figure 2).

Different vaccine adjuvants in HPV vaccine are crucial for the effectiveness of the vaccine immunity as adjuvants facilitate higher levels of, and longer-lasting, immunity (13, 45). However, challenges may arise on the molecular genetic interactions between these adjuvants and the host immune response genes. Table 1, summarizes the immune response genes studied in different childhood vaccines, their outcomes in different populations, and the comparisons between HPV vaccine and the different vaccine adjuvants used.

**Table 1:** Summary of common immune response genes studied in different vaccines and the adjuvants used to facilitate the immune responses in different populations with their comparisons to the HPV vaccine.

**Gene**	**Immune cells for expression**	**Function/Role**	**Study population and outcome**	**Studied vaccine**	Comparison to HPV vaccine^*^	**References**
*BTNL2*	Major Histocompatibility Complex	Immune surveillance	Asian, variants associated with increased vaccine response	Hepatitis B	Similar, VLPs, same adjuvant, alum	(20, 46)
*CD46*	C3-like protein	Inhibitory complement receptor	African and American, variants associated with reduced antibody response	Measles	Different, no alum adjuvant, live attenuated virus	(47, 48)
*FOXP1*	FOX transcription factor families	DNA-binding-and protein-protein binding-domains	Asian, variants associated with increased vaccine response	Hepatitis B	Similar, VLPs, same adjuvant, alum	(20, 46)
*HLA*	Major Histocompatibility Complex	Present peptides to T cells	European, variants associated with persistence of immunity	Tetanus toxoid	Similar (inactive), same adjuvant, alum or alum + MPL	(5)
			Asian, variants associated with both vaccine response and non-response	Hepatitis B	Similar, VLPs, same adjuvant, alum	(20, 49, 50)
			American and European, variants associated with both increased and reduced antibody responses	Rubella	Different, no alum adjuvant, live attenuated virus	(40, 51)
			American, variants associated with both humoral and cellular immunity	Measles	Different, no alum adjuvant, live attenuated virus	(20, 52)
			African, activated HLA-DR^+^ CD4^+^ T cells associated with subsequent risk of TB disease	BCG	Different, no alum adjuvant, live attenuated virus	(42)
*IL12B*	Activated macrophages	Long-term protection to an intracellular pathogen	American, variants associated with increased antibody response	Measles	Different, no alum adjuvant, live attenuated virus	(41, 53)
*ITGAL*	All leukocytes	Leukocyte intercellular adhesion, lymphocyte co-stimulatory signaling	African, variants associated with increased vaccine response	Hepatitis B	Similar, VLPs, same adjuvant, alum	(54)
*PVR*	Plasma cells (Immunoglobulin)	Protein coding, cell adhesion molecule	American, variants associated with reduced antibody levels	Rubella	Different, no alum adjuvant, live attenuated virus	(55)
*RARB*	Thyroid-steroid hormone receptors	Protein coding	American, variants associated with reduced antibody levels	Rubella	Different, no alum adjuvant, live attenuated virus	(55)
			American, variants associated with increased antibody response	Measles	Different, no alum adjuvant, live attenuated virus	(14)
*RIG1*	Interferon-gamma	Initiate antiviral immunity of RNA virus infection, regulation of immune response	American, variants were associated with antibody variations in Caucasians	Measles	Different, no alum adjuvant, live attenuated virus	(14)
*SIRP*	Myeloid dendritic cells, T-cells and a subset of B cells	Innate immune recognition and regulation, enhances antigen-specific T cell proliferation	European, variants associated with persistence of immunity	Haemophilus influenza type b	Different, conjugate but same adjuvant, alum	(5)
			European, variants associated with persistence of immunity	Group C Meningococcal	Different, can be conjugated or unconjugated	(5)

*- The interactions between vaccine adjuvants, host immunogenetic and immunogenomic polymorphisms are purely of authors' opinions*.

* *-“similar” means the “studied vaccine” has a resemblance to the HPV vaccine by containing the same adjuvant (which is then named after. e.g., Alum) and/or the same mode of action. e.g., Viral-like particles*.

*- “different” means the “studied vaccine” differs from the HPV vaccine either by NOT containing the same adjuvant or by having a different mode of action e.g., live attenuated virus*.

*- Alum and/or alum+MPL are among the types of adjuvants HPV vaccine contains*.

HLA polymorphisms provide diversity in host immune responses to multiple, varied, and frequently changing pathogen antigens. This high level of polymorphism may present a challenge to the development of an effective HPV vaccine with life-long POI against HPV infection. Published information relating to the different influence of HLA genes on immune responses according to the antigen and cytokine pathways, suggest that HLA genes vary in their capacity to stimulate HPV immune responses. However, non-HLA genes also play a more important role in HPV immune responses. To quote Mentzer et al. (20); “The HLA associations observed with some vaccines' immune responses, make it clear and intuitive that HLA genes are involved at a key biological level.”

## Proposed Research Questions That Address the HPV Vaccine Poi According to Host Molecular Genetic Variations in Different Populations

### Primary Research Questions and Opportunities

- Is there any association between ethnic origin and impaired HPV vaccine immune responses, POI and adverse vaccination events? **Rationale:** Because the available data is limited for HPV vaccine, and data for other vaccines is mostly based on Caucasian populations and it is unclear how this would relate to other ethnic groups in Sub-Saharan Africa (5).
- Is there any correlation between HPV vaccine VLPs seropositivity and copy numbers, and immunogenicity and POI in different ethnic groups? **Rationale:** As in (i) above, it is unclear how this would relate to other ethnic groups in Sub-Saharan Africa (5).
- Do serum antibody levels post HPV vaccination correlate with adverse vaccination events and the POI in different populations? **Rationale:** Apart from differences in host genetic background, malnutrition and pre-existing infectious diseases like Malaria, human immunodeficiency virus (HIV), and worm infestations may affect the effectiveness of vaccines (17, 56).
- What are the acute and chronic adverse events of HPV vaccination, according to variations in host immune response genes and the number of vaccine doses? **Rationale:** Host molecular genetic variations may influence HPV vaccine adverse events, and these effects may be compounded by the number of vaccine doses (57, 58).
- What are the acute and chronic adverse events of HPV vaccination according to different HPV vaccine dosage regimens in different populations? (Common vs. uncommon adverse events). **Rationale:** In addition to the factors in (iv) above, the genetic differences between populations may change the incidence and severity of vaccine adverse events (58).
- What is the difference between, and within, vaccine cohorts with respect to primary or secondary HPV vaccine failure? **Rationale:** Host molecular genetic variations in the same population or between different populations may have a profound effect on the vaccine effectiveness (25).

### Secondary Research Questions and Opportunities

- Is there a value in examining individualized genetic markers for HPV vaccine immune responses in order to prove their long-term clinical utility? **Rationale:** Individualized genetic markers for HPV vaccine POI may predict the immune responses to vaccination, and may give a clear picture if the HPV vaccine boosters will be needed in some individuals (31).
- What are the molecular genetic determinants for HPV vaccine immunogenicity and effectiveness in different populations? **Rationale:** The individual immune responses to vaccination may vary between populations (29, 32).
- Is there an opportunity for the future use of trans-ethnic HPV vaccine cohorts? **Rationale:** The availability of vaccine cohorts from distinct populations will make it easier to study molecular genetic variations which may influence individual HPV vaccine effectiveness or may induce vaccine failure in the future (5).
- Is there a place for GWAS of common molecular genetic variations and HPV vaccine immune responses, and POI in different populations? **Rationale:** NGS technologies with WGS or WES of all children at birth may facilitate the mapping of individual risks for genetic diseases, predictive, and personalized genetic care in the future, which has already been foreseen in the United Kingdom as a “genomic revolution” (59).
- Can GWAS for immune persistence of HPV vaccine due to HLA variations detect relationships between:
  - Single dose vaccine vs. 2 and 3 doses vaccines? **Rationale:** Number of doses may influence the persistence of HPV immunity (60).
  - Responses to pre-existing infections in infectious disease endemic areas like Sub-Saharan Africa vs. America or Europe? **Rationale:** Pre-existing infectious diseases like Malaria, HIV, and worm infestations may affect the effectiveness of vaccines (17, 56).
- Can longitudinal studies be performed to assess HPV vaccine efficacy vs. effectiveness according to common molecular genetic variations in different populations. **Rationale:** Molecular genetic variations exert distinct influences on antigen specific immune responses (29, 32).
- Are there any host molecular genetic variants linked to interfere with HPV antibody levels after vaccination in some women in the future? **Rationale:** Host molecular genetic variations may exert distinct influences on antigen specific immune responses (29, 32).
- Can host *HLA* variations across populations offer the same immune responses to VLPs in the HPV vaccine? **Rationale:** The early immune response signals originate from differing abilities of classical HLA alleles to bind HPV vaccine VLPs during vaccination (29).
- Is it possible to design individualized HPV vaccine boosters according to immunogenicity, and immunogenetic variations to HPV vaccine POI between populations? **Rationale:** Individual molecular genetics may affect the vaccine induced HPV infection POI (41).
- Is it possible to identify vaccinee cohorts between different populations that yield new genetic loci associated with HPV vaccine POI? **Rationale:** Individual molecular genetic variations may affect the vaccine induced HPV infection POI, and these may vary between populations (41).

Overall, these research questions represent works that should be performed in the idealized research environment. Many of these questions could prove difficult to address, but the questions are still worth asking. The value of the research suggested by these questions would, obviously, rely on the correct experimental design and may require large cohorts and properly randomized protocols to answer the questions posed.

## General Discussion and Future Perspectives

We have examined the host molecular genetic factors that may determine HPV vaccine POI in long-term cervical cancer prevention. Sustained HPV vaccine-induced immunity is crucial in order to combat the burden of cervical cancer across populations. We have summarized the current literature, and have suggested future novel concepts in common host molecular genetic variations in immune responses that may impair HPV vaccine POI. We have identified a number of novel research questions and opportunities involving host molecular genetic variations and the HPV vaccine POI for life-long cervical cancer prevention. These research questions should inform future insights for improved HPV vaccine designs based on common host molecular genetic variations: Firstly, knowledge of the host molecular genetic variations described would explain, and further suggest why acute HPV vaccine induced immunity may not persist in all women in different populations. Secondly, common variations in the immune response genes responsible for HPV vaccine VLPs antigen presentation, and immune recognition in the host, may explain why some women have reported adverse events after HPV vaccination. This may be due to the interactions between vaccine adjuvants and immune response genes, similar to previously observations by Anaya et al. (61), Ozawa et al. (58), Palmieri et al. (57), Pellegrino et al. (62), Perricone et al. (63), and Vera-Lastra et al. (64).

In the pre-HPV vaccine era, the vast majority of women, about 90%, had sufficient immunity to contain or clear the HPV infection, naturally. Only a small minority (~10%) of women developed persistence of oncogenic HPV infection and later cervical precancerous lesions (36, 65). Approximately, 32–43% of these cervical precancerous lesions would regress spontaneously without developing into invasive cervical cancer (ICC) (66). Suggesting that most women have no problem coping with oncogenic HPV infection. This leads to the question: “why do we vaccinate all children, and all with equal dose?” It may be too soon to address this question, but this should be the research focus for the near future in order to avoid vaccinating people unnecessarily and with one dosage regimen. In addition, even though the HPV vaccine adds an extra protection against ICC to the small minority, a small number of women may not respond well to the HPV vaccination. In many HPV vaccine clinical trials, the acute immune responses and vaccine efficacy measured could be from those individuals who would in any case, have been able to cope with oncogenic HPV infection (Vast majority), and the at risk minority may still be missed. Moreover, to date there is no report of any child whose HPV vaccine-induced immunity has waned and would benefit from additional booster immunization.

Although several environmental risk factors have been demonstrated to influence the immune responses to vaccines, genetic factors are the major determinants (67). For example, Leo et al. (43) found that “women with genetic risk scores in the bottom 10% of the population were shown to have a risk of cervical neoplasia of <0.17%, compared with an estimated 1% of women with HPV who develop neoplasia. In contrast, women in the highest 10% of genetic risk scores were shown to have a >7.5% risk for developing cervical neoplasia, and women in the top 5% had a 22% risk for developing the disease.”

Identification of the non-HLA genes influencing HPV antibody responses in vaccinated women should aid the development of novel and, improved HPV vaccine, and its adjuvants that act independently of the *HLA* restrictions. To quote Mentzer et al. (20); “if a gene regulating an innate immunity pathway was discovered to be strongly associated with the level of immune responses to HPV vaccine VLP antigen, the addition of an adjuvant known to enhance signaling through that specific pathway might be expected to enhance immunogenicity, and thereby the effectiveness of the vaccine.” This may override the effects of any variants occurring in the HLA genes or pathways in those individuals.

It is therefore important that individuals who have experienced primary or secondary HPV vaccine failures should be well-examined in order to determine whether they also possess different allele frequencies of the risk or protective *HLA* alleles (20). If such molecular genetic differences are scientifically confirmed, it would be necessary to develop trial methods that may study immune responses in these individuals, perhaps through increased dosing, frequency of vaccine administration (boosters), use of a stronger adjuvant” or ultimately through improved HPV vaccine re-design.

Implementing molecular genetic profiling or WGS at a national level may or may not be practical or economical for now, but it may certainly become ethically compelling to target at-risk individuals or population groups in the future in order to bring about the prospect of personalized HPV vaccination. The preventative healthcare strategies may involve universal genotyping of new-borns on a national scale and this may become a reality in the future in an effort to predict disease genetic risk (20, 59). Such a strategy could become particularly relevant for HPV vaccine delivery if it becomes possible to predict vaccine effectiveness and, perhaps equally importantly, reactogenicity (20). This would be a cost-effective way to risk-stratify individuals in the population to enable targeted HPV vaccination, rather than depending upon the national mass administration programmes.

Novel methods of creating vaccine adjuvants with personalized vaccination based on molecular genetic variations, may become useful strategies in settings, where vaccine immune responses are suboptimal resulting in a raised incidence of HPV vaccine failure. A previous study carried out in the UK and the Netherlands by O'Connor et al. (5), on three common childhood vaccines, has suggested why some vaccines would not work with all children across populations. Taking this new evidence into account in HPV vaccination: temporary immune responses, high HPV antibody titers, or immunogenicity for some years may not prevent vaccinated women from developing cervical neoplasia and hence ICC in the long run. This is because the persistence of cervical oncogenic HPV infection is the most important factor in cervical carcinogenesis.

HPV vaccine failures can be studied further by genotyping via WGS or WES. As suggested by O'Connor et al. (5); “NGS approaches are more conducive to the description of rare variants, however, the cost of these approaches often precludes their use.” Moreover, GWAS are warranted in the investigation of common molecular genetic factors associated with HPV vaccine POI in different populations.

Longer-term surveillance in a vaccinated population is needed to identify waning immunity, and to evaluate requirements for booster immunizations to improve HPV vaccine effectiveness. Future genetic studies with respect to *HLA*, would benefit from scientific approaches that move beyond individual allele–disease associations to investigate biologically relevant groups. It is hoped that the identification of genetic markers for HPV vaccine immune responses and the POI could soon prove their clinical utility because the costs for WGS and WES are progressively decreasing.

## Conclusions

Host molecular genetic variations undoubtedly play a major role in HPV vaccine immune responses and the POI. Assessment of an individual's immunogenetic and immunogenomic backgrounds is crucial for the evaluation of HPV vaccine long-term immunogenicity, effectiveness, and safety. Molecular genetic profiling for variations in common immune response genes will improve HPV vaccine POI by enabling targeted vaccination according to individual molecular genetics in a more cost-effective manner. However, it is essential that studies investigating the contribution of host molecular genetic variations to HPV vaccine POI are; carefully planned, interpreted with caution, and account for known and unknown environmental factors. The results of these studies should help us to understand how we can design an improved HPV vaccine that protects every child individually, and only administered to those children who are at higher risk. The ability of current HPV vaccine to generate effective lifetime vaccine-induced immunity requires further research in different populations. Our work adds novel insights to the future of HPV vaccine designs based on individualized genetic risk, common host molecular genetic variations, and HPV vaccine POI in different populations.

## Author Contributions

RC drafted the manuscript, EK and GR contributed equally to the editing and completion of the manuscript.

## Conflict of Interest

The authors declare that the research was conducted in the absence of any commercial or financial relationships that could be construed as a potential conflict of interest.
